# Step detection and energy expenditure at different speeds by three accelerometers in a controlled environment

**DOI:** 10.1038/s41598-021-97299-z

**Published:** 2021-10-08

**Authors:** Ville Stenbäck, Juhani Leppäluoto, Nelli Leskelä, Linda Viitala, Erkki Vihriälä, Dominique Gagnon, Mikko Tulppo, Karl-Heinz Herzig

**Affiliations:** 1 Research Unit of Biomedicine, Medical Research Center, Faculty of Medicine, University of Oulu, and Oulu University Hospital, P.O. Box 5000, 90014 Oulu, Finland; 2 Biocenter Oulu, Oulu, Finland; 3 Optoelectronics and Measurement Techniques Research Unit, Faculty of Information Technology and Electrical Engineering, University of Oulu, Oulu, Finland; 4 Department of Sports and Exercise Medicine, Clinicum, University of Helsinki, Helsinki, Finland; 5 Department of Gastroenterology and Metabolism, Poznan University of Medical Sciences, Poznan, Poland

**Keywords:** Obesity, Diabetes, Metabolic syndrome, Pre-diabetes, Lifestyle modification, Preventive medicine, Disease prevention, Public health

## Abstract

Physical activity (PA) is one of the most efficient ways to prevent obesity and its associated diseases worldwide. In the USA, less than 10% of the adult population were able to meet the PA recommendations when accelerometers were used to assess PA habituation. Accelerometers significantly differ from each other in step recognition and do not reveal raw data. The aim of our study was to compare a novel accelerometer, Sartorio Xelometer, which enables to gather raw data, with existing accelerometers ActiGraph GT3X+ and activPAL in terms of step detection and energy expenditure estimation accuracy. 53 healthy subjects were divided into 2 cohorts (cohort 1 optimization; cohort 2 validation) and wore 3 accelerometers and performed an exercise routine consisting of the following speeds: 1.5, 3, 4.5, 9 and 10.5 km/h (6 km/h for 2nd cohort included). Data from optimization cohort was used to optimize Sartorio step detection algorithm. Actual taken steps were recorded with a video camera and energy expenditure (EE) was measured. To observe the similarity between video and accelerometer step counts, paired samples *t* test and intraclass correlation were used separately for step counts in different speeds and for total counts as well as EE estimations. In speeds of 1.5, 3, 4.5, 6, 9 and 10.5 km/h mean absolute percentage error (MAPE) % were 8.1, 3.5, 4.3, 4.2, 3.1 and 7.8 for the Xelometer, respectively (after optimization). For ActiGraph GT3X+ the MAPE-% were 96.93 (87.4), 34.69 (23.1), 2.13 (2.3), 1.96 (2.6) and 2.99 (3.8), respectively and for activPAL 6.55 (5.6), 1.59 (0.6), 0.81 (1.1), 10.60 (10.3) and 15.76 (13.8), respectively. Significant intraclass correlations were observed with Xelometer estimates and actual steps in all speeds. Xelometer estimated the EE with a MAPE-% of 30.3, activPAL and ActiGraph GT3X+ with MAPE percentages of 20.5 and 24.3, respectively. The Xelometer is a valid device for assessing step counts at different gait speeds. MAPE is different at different speeds, which is of importance when assessing the PA in obese subjects and elderly. EE estimates of all three devices were found to be inaccurate when compared with indirect calorimetry.

## Introduction

Physical activity (PA) is one of the most efficient ways to prevent and treat obesity and its associated diseases worldwide. At least 26 chronic diseases can be influenced or prevented with sufficient PA including conditions ranging from depression, dementia to diabetes and hypertension^1,2^. WHO recommends 150 min of moderate-intensity PA in a week or a combination of moderate and vigorous-intensity PA with additional health benefits arising with increased exercise amounts^3^. This responds approximately 7000–10,000 daily steps^4^. Muscle-strengthening activities should be done twice a week or more. The use of accelerometers enables to objectively assess the subjects’ PA behavior (volume and intensity), but the devices are not equal in their step-detection thresholds, sampling frequency and data processing^5,6^. In the USA, less than 10% of the adult population and 24% in Finland were able to meet the recommendations when accelerometers were used to assess PA habituation^7,8^. Healthy older adults (50 + years old) take 2000 to 9000 daily steps and special populations (older or diseased/disabled) 1200 to 8800 steps^9^. Previously, we have shown that most prediabetic subjects could not reach the PA recommendations and their activity was at a low-intensity level of below 2 METs (or 0.3–0.7 g, speed of 2–3 km/h)^10,11^. For older subjects (65–84 years old), 8000–10,000 daily steps are associated with better metabolic health^12^. Chow and colleagues found that waist-worn accelerometers are significantly more accurate than wrist-worn ones (error percentages 0.1 ± 4.2 and − 12.5 ± 19.2, respectively) in speeds of 5–12 km/h^13^. For wrist-worn devices, higher activity counts have been reported when accelerometer has been worn on dominant wrist vs. the non-dominant one^14^. Certain widely used accelerometers, such as the ActiGraph GT3X, tend to become more accurate in detecting steps with higher speeds of more than 4 km/h^15^. For hip-worn accelerometers, at the speed of 2.4 km/h, errors of 6.0–20% were observed e.g. for ActiGraph GT3X+. Pooled analysis of 9 cohort studies collected between 1986 and 2000 in 34,485 elderly people (> 65 years old) with a gait speed between 1.44 to 5 km/h on average had an age-adjusted lower hazard ratio for death per 0.1-m/s higher gait speed^16^. Identifying the overall PA across the gait spectrum and intensity with emphasis on slow walking speed is of significant importance for formulating relevant PA guidelines for overweight, elderly and special populations with case-specific health outcomes.

In the existing accelerometers, the counting systems as intensity over time differ and no raw data are always available^17^. Uni- and triaxial equipment do not agree in all types of activity^18^. Furthermore, there are no standards towards step recognition, area under the curve, sampling frequency, signal filtering, length of the measurement or wearing site^19^, rendering the obtained results impossible to compare. Limitations in non-wear time detection are recognized as well^20^. Because of these severe limitations of the available accelerometers as black boxes, a waist-worn triaxial accelerometer was manufactured registering accelerations from slow walking to running and jumping.

The aim of this study was to compare this novel accelerometer, Sartorio Xelometer, with the two most commonly used accelerometers in scientific research (ActiGraph GT3X+, activPAL) for its accuracy at different locomotion speed and in EE estimation for its step detection.

## Results

### Step detection

Three accelerometers were used to estimate the number of steps taken and each of them were individually compared with the counted steps in the video camera—recordings in the 5 different speeds (6 in cohort 2) to determine the accuracy of different methods (Table 1, 2). Two cohorts with similar anthropometrics were measured. The data from the optimization cohort (cohort 1) were used to optimize the step detection algorithm and tested in cohort 2. The mean absolute percentage error (MAPE) percentages observed in steps detection resulted from the underestimation of step counts in all three devices. Bland–Altman plots were constructed to visualize the relationship between the mean and difference of actual and estimated steps in each speed for the three devices (Supplementary Figures 1–3, 5–7)^21^. The exact means and 95% limits of agreement for the plots are presented in the Supplementary Tables 1 and 2.

**Table 1:** 35 healthy subjects (optimization cohort 1) participated in the study and performed a 20-min exercise routine with 3 walking (1.5, 3 and 4.5 km/h) and 2 running (9 and 10.5 km/h) speeds. Each speed was recorded for 4 min. The performance was recorded with a video camera and steps calculated afterwards for each speed. Mean absolute percentage error (MAPE) percentages, paired sample *t* test statistics and intraclass correlation (ICC) statistics with 95% CI presented for each device in each speed and for total sum of steps. *Shows statistical significance.

	Speed (km/h)	MAPE-% ± Std. dev.	Paired samples *t* test	95% Confidence interval of the difference			ICC	95% Confidence interval		F test	
			Mean ± Std. dev.	Lower	Upper	Sig. (2-tailed)		Lower	Upper	Value	Sig.
Sartorio	1.5	14.678 ± 14.93	− 10.182 ± 52.027	− 28.63	8.266	0.269	0.389	− 0.237	0.698	1.636	0.085
	3	2.900 ± 3.05	5.545 ± 14.697	0.334	10.757	0.038*	0.910	0.817	0.955	11.08	0.000*
	4.5	2.958 ± 1.734	6.303 ± 14.059	1.318	11.288	0.015*	0.811	0.685	0.896	5.285	0.000*
	9	10.177 ± 9.340	66.545 ± 63.522	44.021	89.069	0.000*	0.846	0.688	0.924	6.481	0.000*
	10.5	18.409 ± 10.645	128.563 ± 80.414	99.57	157.555	0.000*	0.177	− 0.788	0.564	1.133	0.363
	Total	8.100 ± 5.930	194.576 ± 155.184	139.55	249.602	0.000*	− 0.293	− 1.649	0.369	0.773	0.761
ActivPAL	1.5	6.550 ± 7.685	14.030 ± 21.708	6.333	21.727	0.001*	0.885	0.767	0.943	8.705	0.000*
	3	1.587 ± 2.561	2.394 ± 10.377	− 1.286	6.073	0.194	0.953	0.904	0.977	21.076	0.000*
	4.5	0.805 ± 1.483	2.152 ± 6.783	− 0.254	4.557	0.078	0.973	0.946	0.987	37.376	0.000*
	9	10.603 ± 9.758	69.909 ± 68.533	45.608	94.21	0.000*	− 1.389	− 3.836	− 0.18	0.419	0.992
	10.5	15.755 ± 11.801	100.606 ± 99.704	65.253	135.959	0.000*	0.791	0.577	0.897	4.787	0.000*
	Total	7.866 ± 5.543	190.818 ± 146.453	138.888	242.748	0.000*	0.567	0.123	0.786	2.309	0.010*
ActiGraph	1.5	96.931 ± 5.299	254.914 ± 30.099	244.574	265.253	0.000*	0.188	− 0.608	0.59	1.232	0.273
	3	34.686 ± 17.185	129.257 ± 66.809	106.307	152.207	0.000*	− 0.017	− 1.016	0.486	0.983	0.520
	4.5	2.127 ± 3.029	8.4 ± 14.235	3.509	13.290	0.001*	0.877	0.756	0.938	8.12	0.000*
	9	1.956 ± 4.957	1.485 ± 34.713	− 10.438	13.410	0.802	0.68	0.366	0.838	3.123	0.001*
	10.5	2.989 ± 4.125	2.257 ± 37.344	− 10.571	15.085	0.723	0.975	0.951	0.988	40.495	0.000*
	Total	16.694 ± 4.429	397.942 ± 110.348	360.036	435.848	0.000*	0.827	0.657	0.913	5.776	0.000*

**Table 2:** Energy expenditure (EE) estimates from three devices compared with indirect calorimetry (IC) in the optimization cohort 1. Mean absolute percentages error (MAPE) percentages, paired sample *t* test statistics and intraclass correlation (ICC) statistics with 95% CI presented for each device for total 20 min of testing. *Shows statistical significance.

	MAPE-% ± Std. dev.	Paired samples *t* test	95% Confidence interval of the difference			ICC	95% Confidence interval		F test	
		Mean ± Std. dev.	Lower	Upper	Sig. (2-tailed)		Lower	Upper	Value	Sig.
Sartorio	30.324 ± 16.360	− 1.337 ± 0.678	− 1.590	− 1.084	0.000*	0.209	− 0.663	0.623	1.264	0.266
ActivPAL	20.545 ± 10.766	1.239 ± 1.134	0.827	1.655	0.000*	0.175	− 0.712	0.602	1.211	0.301
ActiGraph	24.319 ± 9.596	1.186 ± 0.680	0.917	1.455	0.000*	0.471	− 0.162	0.759	1.889	0.056

### Optimization cohort 1

**Figure 1:**
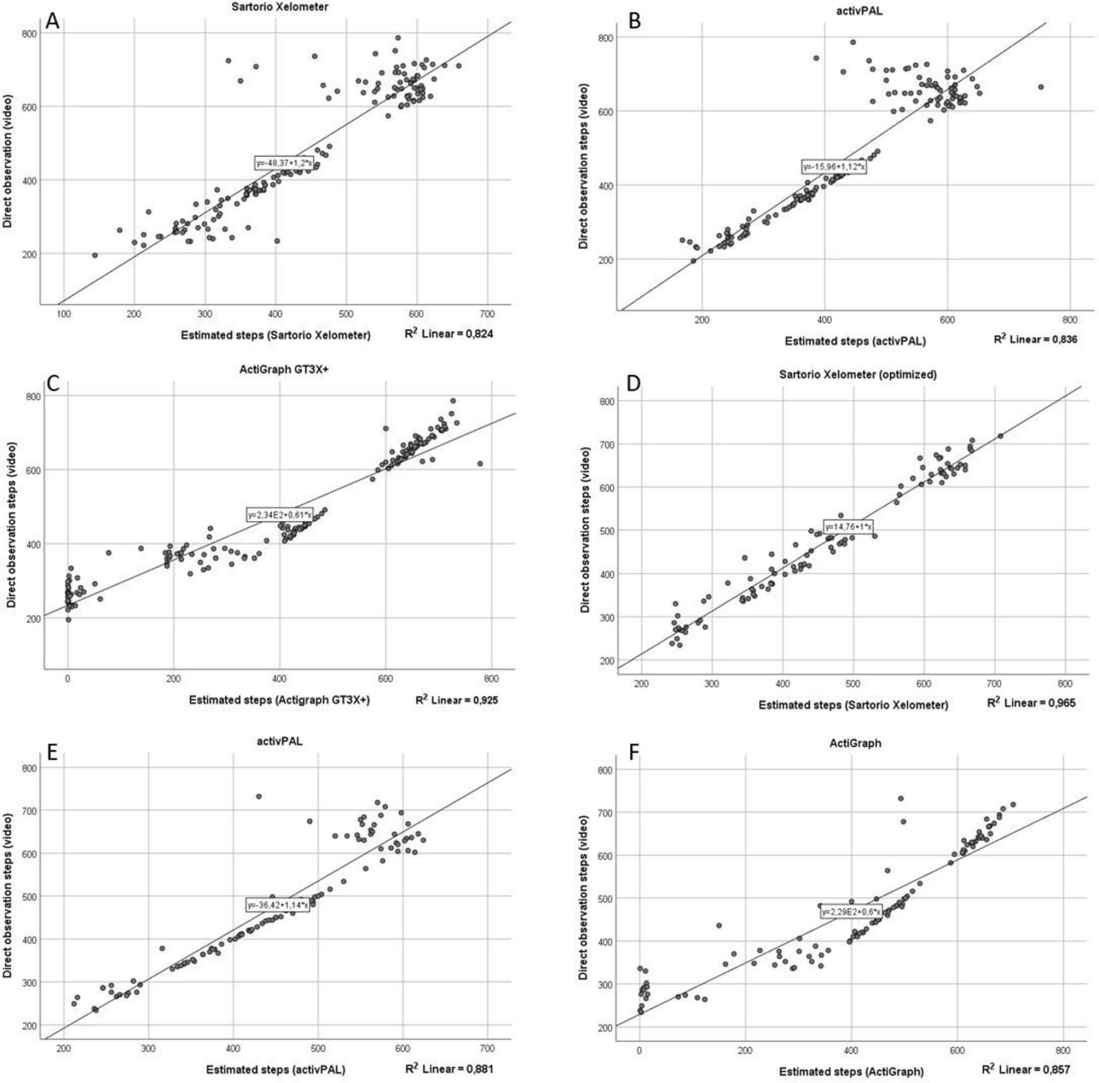
The regression plots between different accelerometer detected steps and direct measurement. All five speeds have been plotted separately. (**A**–**C**) Optimization cohort 1. (**D**–**F**) Validation cohort 2. (**A**) Sartorio Xelometer R^2^ = 0.824. (**B**) activPAL R^2^ = 0.836. (**C**) ActiGraph GT3X+ R^2^ = 0.925. (**D**) Sartorio R^2^ = 0.965. (**E**) activPAL R^2^ = 0.881. (**F**) ActiGraph GT3X+ R^2^ = 0.925.

The Sartorio Xelometer was more inaccurate in the very high running speeds (Table 1). In running speeds (9, 10.5 km/h) the MAPE percentages were 10.2 and 18.4, respectively with a significant difference with the recorded and counted steps. In walking speeds (1.5, 3 and 4.5 km/h) 14.7, 2.9 and 3.0% of MAPE were observed with a significant difference between video camera-observations and accelerometer estimates in 3 and 4.5 km/h. The total steps taken estimated with Xelometer differed 8.1% on average with the counted steps. The mean difference in the Bland–Altman plot (Supplementary Figure 1) was 79.6 (95% CI lower: − 132.0, upper: 291.2) and the mean differences for the individual speeds ranged between − 10.2 and 128.6 (95% CI lower: − 67.5 to 195.6, upper: 34.5 to 315.1) (Supplementary Table 1). R^2^ between the actual and device-estimated steps was 0.824 when considering all the different speeds individually (Fig. 1A). The intraclass correlations were significant in speeds between 3 and 9 km/h with good or excellent correlation coefficients (ICC > 0.75 and > 0.90, respectively).

The thigh-worn activPAL performed most accurately in the walking speeds of 1.5, 3 and 4.5 km/h speeds, with the MAPE percentages 6.6, 1.6 and 0.8, respectively (Table 1). In the walking speeds, a significant difference between the observed and estimated step counts was detected in 1.5 km/h speed but not with 3 or 4.5 km/h speeds (p = 0.001, 0.194 and 0.078, respectively). While running (9 and 10.5 km/h), MAPE percentages were 10.6 and 15.8, respectively. Statistical differences between actual and meter-estimated steps were found in both running speeds. When all speeds evaluated together, MAPE percentage was 7.9 with a significant difference with the video recorded step counts. Regression analysis resulted in R^2^ of 0.836 when all speeds were individually considered (Fig. 1B). Statistically significant intraclass correlations were observed in all speeds except in 9 km/h. The mean difference in the Bland–Altman plot (Supplementary Figure 2) was 38.3 (95% CI lower: − 94.5, upper: 171) for all speeds and during walking ranged between 2.4 and 14.0 (95% CI lower: − 2.9 to 28.5, upper: 22.7 to 56.6) and between 69.9 and 100.6 (95% CI lower: − 94.8 and − 64.4, upper: 296.0 and 204.2) during running (Supplementary Table 1).

The ActiGraph GT3X+ performed well in higher exercise speeds (4.5, 9 and 10.5 km/h) with mean absolute error (MAPE) percentages of 2.1, 2.9 and 3.0, respectively. In the paired samples *t* test a significant difference between video-camera observed and ActiGraph-estimated steps was not observed in the running speeds (9 and 10.5 km/h, p = 0.802 and 0.723, respectively). In walking speeds (1.5 and 3 km/h), the GT3X+ was less accurate with MAPE percentages of 96.9 and 34.7, respectively and a significant difference was observed in step counts between the methods (Table 1). There was a significant difference between the observation methods in the total steps taken during the 20-min period with MAPE percentage of 16.7. Significant intraclass correlations were observed in 4.5, 9, 10.5 km/h and total steps taken. The R^2^ for the regression between individual actual and estimated steps was 0.925 (Fig. 1C). For the Bland–Altman plot (Supplementary Figure 3) the mean difference for all speeds was 79.3 (95% CI lower: − 133, upper: 291.6). The differences for the individual speeds were high on the two lowest speeds (means: 254.9 and 129.3, 95% CI lower: 195.9 and 1.7, upper: 313.9 and 260.2), and lower for the three higher ones, between 2.3 and 14.2 (95% CI lower: − 19.5 to − 70.9, upper: 36.3 to 82.3) (Supplementary Table 1).

### EE estimates

**Figure 2:**
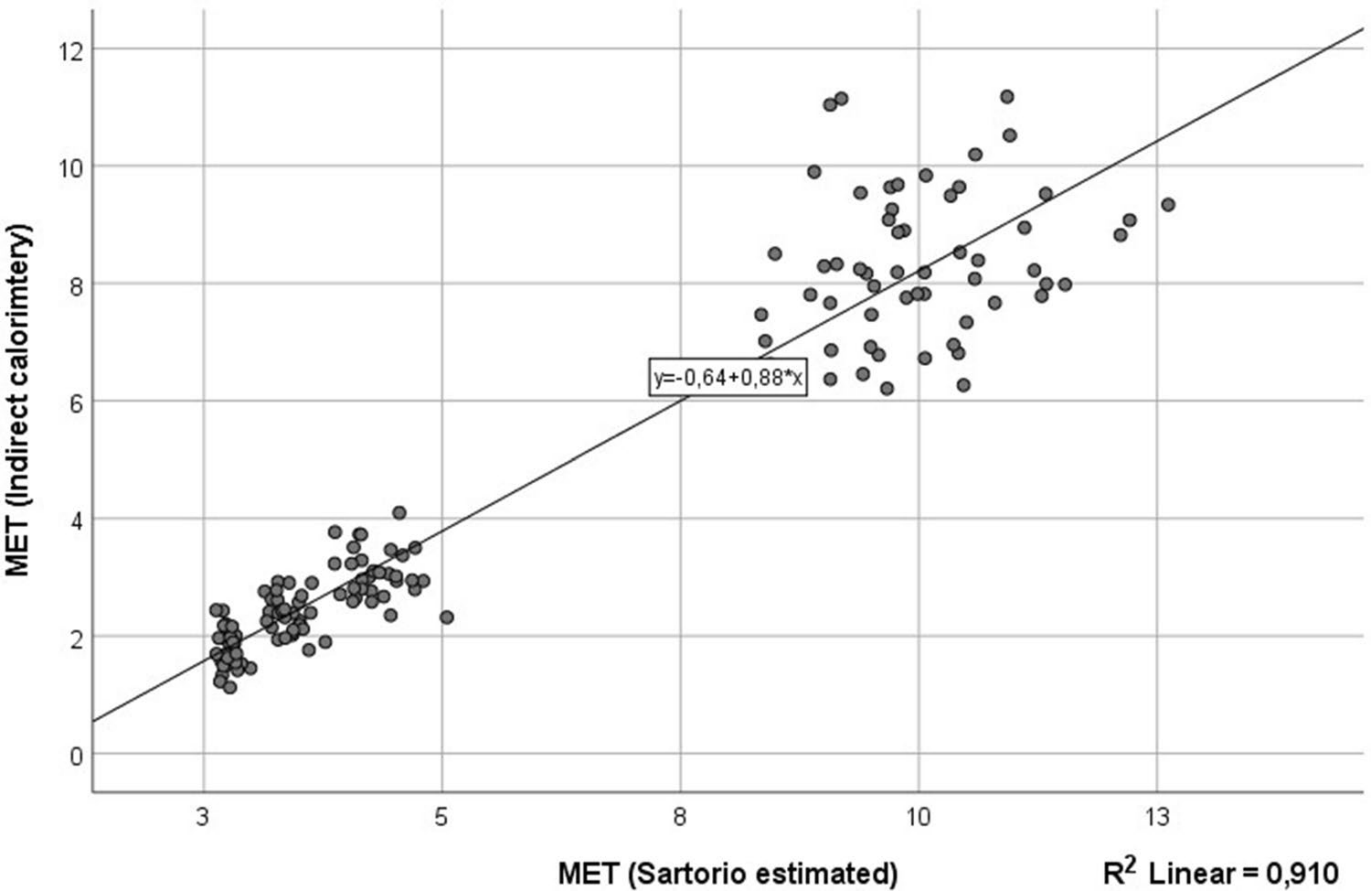
The regression plot between indirect calorimetry MET and Sartorio Xelometer—estimated MET. All five speeds have been plotted separately. R^2^ = 0.910.

All three devices were used to assess EE (Table 2). The MAPE percentages for activPAL, ActiGraph and Sartorio were 20.5, 24.3 and 30.3, respectively. Significant differences were observed between the indirect calorimetry (IC) measured EE and accelerometer-estimated EE in all devices. None of the accelerometers EE estimates correlated significantly with the IC measured EE. To assess the EE estimates more closely, we did a comparison between IC measured EE and Sartorio Xelometer-estimated EE as METs for each five speeds (Supplementary Figure 4, Supplementary Table 2). For walking (1.5, 3 and 4.5 km/h), the MAPE percentages 63.0, 46.0 and 42.9, respectively (Table 3). For running (9 and 10.5 km/h), MAPE %’s were 29.4 and 18.4, respectively. The R^2^ between IC METs and accelerometer METs was 0.910 (Fig. 2). Significant differences were observed in all speeds between the accelerometer-estimated and IC EE. No significant intraclass correlations were found.

**Table 3:** Sartorio Xelometer MET-estimation in different speeds. 35 healthy subjects (optimization cohort 1) participated in the study and performed a 20-min exercise routine with 3 walking (1.5, 3 and 4.5 km/h) and 2 running (9 and 10.5 km/h) speeds. Each speed was recorded for 4 min. The energy expenditure was recorded using indirect calorimetry and EE was calculated for each speed. IC calculated EE was compared with Sartorio Xelometer—estimated EE in MET. Mean absolute percentage error (MAPE) percentages, paired sample *t* test statistics and intraclass correlation (ICC) statistics with 95% CI presented for each device in each speed and for total sum of steps. *Shows statistical significance.

	Speed (km/h)	MAPE-% ± Std. dev.	Paired samples *t* test	95% Confidence interval of the difference			ICC	95% Confidence interval		F test	
			Mean ± Std. dev.	Lower	Upper	Sig. (2-tailed)		Lower	Upper	Value	Sig.
Sartorio	1.5	62.973 ± 31.479	− 1.004 ± 0.359	− 1.138	− 0.869	0.000*	− 0.249	− 1.624	0.405	0.800	0.723
	3	46.038 ± 23.020	− 1.042 ± 0.393	− 1.189	− 0.895	0.000*	− 0.262	− 1.651	0.399	0.792	0.732
	4.5	42.877 ± 24.560	− 1.260 ± 0.567	− 1.472	− 1.048	0.000*	− 0.520	− 2.192	0.276	0.658	0.867
	9	29.410 ± 15.911	− 2.012 ± 1.292	− 2.495	− 1.529	0.000*	0.380	− 0.303	0.704	1.612	0.102
	10.5	18.484 ± 14.458	− 1.355 ± 1.431	− 1.899	− 0.810	0.000*	0.230	− 0.639	0.638	1.298	0.246
	Total	30.325 ± 16.360	− 1.337 ± 0.678	− 1.590	− 1.084	0.000*	0.209	− 0.662	0.623	1.263	0.266

### Validation cohort 2

After the optimization of the Sartorio step detecting algorithm, a validation cohort 2 was investigated (Table 4). At 1.5 km/h MAPE-% was 8.1 with a significant difference in step numbers between the direct measurement and Xelometer-estimated steps (Table 5). At the additional walking speeds (3, 4.5 and 6 km/h) the MAPE-%s were 3.5, 4.3 and 4.2, respectively. No significant differences between direct observation and estimated step counts were detected. At running speeds (9 and 10.5 km/h), the MAPE-%’s were 3 and 4.8, respectively. The MAPE-% for total 24-min step count was 2.7. At 10.5 km/h and total step counts, significant differences were observed between the direct observation and estimated steps. Significant intraclass correlations were detected in all speeds and the total step numbers, with correlation coefficients between 0.62 and 0.99. The R^2^ for the regression between the direct observation and estimated steps was 0.965 (Fig. 1D). In the Bland–Altman plot (Supplementary Figure 5), the mean difference for all speeds was 73 (95% CI lower: − 33.2, upper: 179.2). At the individual speeds, the mean differences ranged between 3.6 and 30.7 (95% CI lower: − 44.2 to 13.2, upper: 50.6 to 74.6) (Supplementary Table 3).

**Table 4:** Study population characteristics (n = 54). *BMI* body mass index, *SMM* skeletal muscle mass.

	Optimization cohort 1	Validation cohort 2
Sex	13 male, 22 female	12 male, 7 female
Age (years)	30.6 ± 9.2	33.5 ± 8.3
Height (cm)	172.5 ± 9.1	173.7 ± 9.9
Weight (kg)	68.5 ± 10.9	74.4 ± 13.0
BMI	23.0 ± 2.5	24.6 ± 3.24
SMM-% (impedance)	44.5 ± 5.1	43.4 ± 5.6
Fat-% (impedance)	22.9 ± 2.5	22.9 ± 9.0

**Table 5:** 19 healthy subjects (validation cohort 2) participated in the study and performed a 24-min exercise routine with 4 walking (1.5, 3, 4.5 and 6 km/h) and 2 running (9 and 10.5 km/h) speeds. Each speed was recorded for 4 min. The performance was recorded with a video camera and steps calculated afterwards for each speed. Mean absolute percentage error (MAPE) percentages, paired sample *t* test statistics and intraclass correlation (ICC) statistics with 95% CI presented for each device in each speed and for total sum of steps. *Shows statistical significance.

	Speed (km/h)	MAPE-% ± Std. dev.	Paired samples *t* test	95% Confidence interval of the difference			ICC	95% Confidence interval		F test	
			Mean ± Std. dev.	Lower	Upper	Sig. (2-tailed)		Lower	Upper	Value	Sig.
Sartorio	1.5	8.101 ± 6.538	19.588 ± 26.884	5.766	33.411	0.008*	0.619	− 0.052	0.862	2.624	0.031*
	3	3.482 ± 4.697	3.588 ± 25.115	− 9.325	16.501	0.564	0.624	− 0.039	0.864	2.656	0.029*
	4.5	4.343 ± 3.638	7.529 ± 25.666	− 5.667	20.726	0.244	0.742	0.287	0.907	3.873	0.005*
	6	4.184 ± 3.073	6.176 ± 25.606	− 6.989	19.342	0.335	0.917	0.772	0.970	12.104	0.000*
	9	3.060 ± 3.445	10.824 ± 20.926	0.064	21.582	0.049	0.981	0.948	0.993	53.292	0.000*
	10.5	4.790 ± 3.079	30.714 ± 23.233	17.300	44.128	0.000*	0.781	0.317	0.930	4.561	0.005*
	Total	2.742 ± 1.837	73.000 ± 55.861	44.279	101.721	0.000*	0.990	0.972	0.996	97.507	0.000*
ActivPAL	1.5	5.642 ± 5.993	13.111 ± 18.917	3.704	22.519	0.009*	0.919	0.783	0.970	12.318	0.000*
	3	0.588 ± 0.959	− 0.833 ± 4.148	− 2.896	1.229	0.406	0.994	0.983	0.997	160.586	0.000*
	4.5	1.121 ± 2.360	3.333 ± 12.852	− 3.058	9.725	0.286	0.936	0.829	0.976	15.621	0.000*
	6	1.163 ± 1.885	2.667 ± 13.771	− 4.182	9.515	0.423	0.969	0.917	0.988	32.117	0.000*
	9	10.270 ± 6.559	63.557 ± 46.902	40.232	86.879	0.000*	0.866	0.641	0.949	7.450	0.000*
	10.5	13.790 ± 10.419	93.867 ± 77.936	50.707	137.026	0.000*	− 3.500	− 12.404	− 0.510	0.222	0.996
	Total	5.619 ± 3.870	160.056 ± 122.323	99.226	220.886	0.000*	0.935	0.826	0.975	15.393	0.000*
ActiGraph	1.5	87.397 ± 16.668	246.389 ± 53.910	219.58	273.198	0.000*	0.297	− 0.880	0.736	1.421	0.237
	3	23.071 ± 16.909	87.000 ± 71.296	51.545	122.455	0.000*	− 0.311	− 2.504	0.509	0.762	0.708
	4.5	2.332 ± 4.597	9.278 ± 24.190	− 2.752	21.307	0.122	0.718	0.246	0.894	3.544	0.006*
	6	3.112 ± 7.410	11.833 ± 39.825	− 7.971	31.638	0.224	0.813	0.500	0.930	5.346	0.000*
	9	2.560 ± 5.959	13.611 ± 42.264	− 7.406	34.629	0.190	0.910	0.759	0.966	11.100	0.000*
	10.5	3.750 ± 7.813	20.067 ± 61.969	− 14.251	54.384	0.230	− 0.604	− 3.777	0.461	0.623	0.806
	Total	14.121 ± 6.290	384.833 ± 164.711	302.924	466.742	0.000*	0.920	0.785	0.969	12.462	0.000*

In the validation cohort, the results for the activPAL and ActiGraph were similar to the optimization cohort (Fig. 1E,F). At 6 km/h, both devices had low MAPE-%s of 1.2 and 3.1, respectively and no significant differences between estimated and directly observed steps were found (Table 5). The R^2^’s of the regressions between direct observation and estimated steps were 0.88 and 0.86, respectively.

## Discussion

We compared the novel Xelometer for step detection in a controlled environment at different speeds and compared it to the two most commonly used accelerometers, ActiGraph GT3X+ and activPAL. Since the step detection of Sartorio Xelometer was found to be inaccurate in slow walking (1.5 km/h) and running speeds (9, 10.5 km/h), we decided to optimize the algorithm with machine learning by using the data from the optimization cohort 1 and to test the optimized algorithm in the validation cohort 2. The protocol in optimization cohort 1 included walking (1.5, 3 and 4.5 km/h or 25, 50 and 75 m/min) and running (9 and 10.5 km/h or 150 and 175 m/min). In addition, the speed of 6 km/h (or 100 m/min) was included in the protocol of the validation cohort 2. Our special interest was on slow walking speeds (1.5 and 3 km/h) since in the elderly and obese population these are the most frequent walking speeds. The optimization of the Sartorio step detection algorithm improved the accuracy on slow walking speed (1.5 km/h) and while running (9 and 10.5 km/h). There was reduction of the correlation coefficients in speeds of 1.5–4.5 km/h but the correlations became significant. All three accelerometers had their optimal speed range and the accuracy varied between devices. After optimizing the step detection algorithm, the Xelometer had the best overall performance in the validation cohort 2 with the total count MAPE percentage of 2.7, compared to ActiGraphs and activPALs 14.1 and 5.6, respectively. The most accurately Xelometer performed at speeds of 3 and 9 km/h. In walking speeds, activPAL was the most accurate device before Sartorio, while ActiGraph GT3X+ did not detect most of the steps. At running speed, the ActiGraph GTX3+ performed the most accurately before Sartorio and activPAL. The intraclass correlations support these findings showing good and significant (> 0.75) or excellent (> 0.90) correlation coefficients for activPAL in all speeds except 10.5 km/h, ActiGraph GTX3+ while running and walking at or over 4.5 km/. For the optimized Sartorio Xelerometer, the correlations were significant in all speeds with good or excellent correlations for the overall step number and in speeds of 4.5–10.5 km/h. Importantly, the Xelometer’s performance was the most stable throughout the protocol with all MAPE-%’s 3.1–4.3, except the 8.1 for 1.5 km/h.

The MAPE%s observed in this study for activPAL and GT3X+ are similar for those published by Feito and colleagues in 2012, where they showed that activPAL error-% are low in speeds of 2.4–5.6 km/h, while GT3X+ becomes accurate in detecting steps with speeds higher than 4 km/h (67 m/min) and significantly underestimates the step counts at lower speeds^15^. At a speed of 3.2 km/h, a 40% error has been reported for the GT3X+, which then diminishes with increasing speed^22^. We did not use ActiGraph’s low frequency extension, since discrepancies have been reported while applying it in overall step detection in free-living conditions^23,24^. Our findings are also in line with Ryan and colleagues, who showed accurate step detection for activPAL in speeds between 3.24 and 6.4 km/h^25^. When selecting a suitable method for PA measurement, the properties of the accelerometers should be considered. If the main interest is in studying older or more sedentary populations, a device more accurate at the lower spectrum of locomotion would be needed.

Based on these results, the Xelometer’s step detection is a valid method to observe subject’s PA volume. In comparison with the two commonly used and validated accelerometers ActiGraph GT3X+ and activPAL, the Xelometer performed equally throughout the protocol (MAPE% 2.7, ICC 0.99) and thus suitable specially to observe the PA level.

All devices performed poorly estimating EE. activPAL estimated the EE closest with a MAPE of 20.5.2%, while Sartorio Xelometer and ActiGraph GT3X+ showed more inaccurate estimations with MAPE percentages of 30.3 and 24.3, respectively. Standard deviations of 9.6–16.4% support the inaccuracy statement. Moreover, ICCs showed no significant correlations with IC measurements in any of the three devices. Calculations of Sartorio Xelometer’s individual EE estimates for each speed showed higher inaccuracies on lower speeds with enhanced accuracy with higher speeds. Both activPAL and ActiGraph GT3X+ underestimated the EE, while an overestimation was observed for Sartorio Xelometer EE. Our error percentages are similar of those reported by Calabró and colleagues with an underestimation of 22.2% and 25.5% for activPAL and ActiGraph GT3X+, respectively^26^. Similar error percentages for ActiGraph GT3X+ but not for activPAL were reported (21.2 and 9.3, respectively) by Alberto and colleagues in light-intensity PA^27^. These results suggest that accelerometer EE estimates are inaccurate and their use in research should be carefully considered.

In our study we need to consider limitations. Our subjects were healthy volunteers with a BMI less than 27 (Optimization cohort 1 23.0 ± 2.5, Validation cohort 2 24.6 ± 3.24). The results observed in this population may not be translatable to a cohort with different anthropometrics and PA capabilities. We also examined the function of the devices in a controlled environment. The strengths of this study include video camera—recorded true steps, both sexes as subjects and the use of two already validated accelerometers.

## Materials and methods

We recruited healthy 54 subjects amongst the students and faculty members of University of Oulu and other City of Oulu institutions (Table 4). The subjects were divided into two cohorts (35 subjects for the optimization cohort 1 and 19 subjects for the validation cohort 2) based on the order of their sign-up. Validation cohort 2 was recruited after the analysis of optimization cohort 1. The study was approved by the ethical committee of the Northern Ostrobothnia Hospital District and was executed in line with the National legislation, guidelines and the Declaration of Helsinki. Written informed consent was given by subjects in accordance with the Declaration of Helsinki.

Subjects in the optimization cohort (n = 35) were asked to fast and retain from strenuous exercise, coffee and nicotine at least 14 h before the study visit. Height and weight were measured in centimeters with one decimal accuracy. BMI was calculated as weight (kg) per height (m) squared. Body composition was determined using bioimpedance with InBody 720. Oxygen uptake and carbon dioxide production were recorded by indirect calorimetry (IC) using Medikro model 909 Ergospirometer^28–30^. The device was calibrated before every subject for volume and gas concentrations. For gas calibration a mixture of oxygen (15%), carbon dioxide (5%) and nitrogen (80%) was used. The subjects had an overnight fast. Resting metabolic rate (RMR) was recorded in a supine position until the levels plateaued at least for 10 min. Last 5 min of the measurement were used to calculate basal metabolic rate (RMR). Respiratory exchange ratio (RER) was monitored to stay within 0.7 and 0.99 during the RMR measurement. Metabolic rate was calculated using the Weir equation: metabolic rate (kcal/day) = 1.44 (3.94 VO^2^ + 1.11 VCO^2^). Since RMR was measured in a supine position and not while sitting, a conversion factor of 7% was used to correct RMR values for postural effect according to Newton et al.^31^. Corrected RMR was used as a level of 1 metabolic equivalent (MET) for further IC analysis for calculating the reference values for accelerometer estimates.

After the baseline measurements subjects were asked to perform an exercise routine of 20 min on a treadmill (OJK 2, Telineyhtymä, Kotka, Finland), which consisted of five 4-min walking and running periods. The speeds were 1.5, 3, 4.5, 9 and 10.5 km/h, respectively. The lower speeds were chosen to correspond the walking speeds in daily behavior of older and diseased subjects. People over 60-years move at a speed of 4.2 km/h on average and prediabetics subjects have been shown to move mainly on speeds between 2–3 km/h^10,32^. The acceleration to the next speed took 5–10 s and was included in the beginning of each 4-min period. A video camera was set up and recorded the subject’s moving feet during the exercise. These videos were used to calculate the actual step counts. Two persons counted the steps from the video^33^. This method of direct observation was chosen to reduce observational error. Oxygen consumption and carbon dioxide production were monitored throughout the exercise protocol to calculate the PA energy expenditure. The total EE for the 20-min protocol was calculated by assessing the EE with Weir equation for every minute and adding them together.

**Figure 3:**
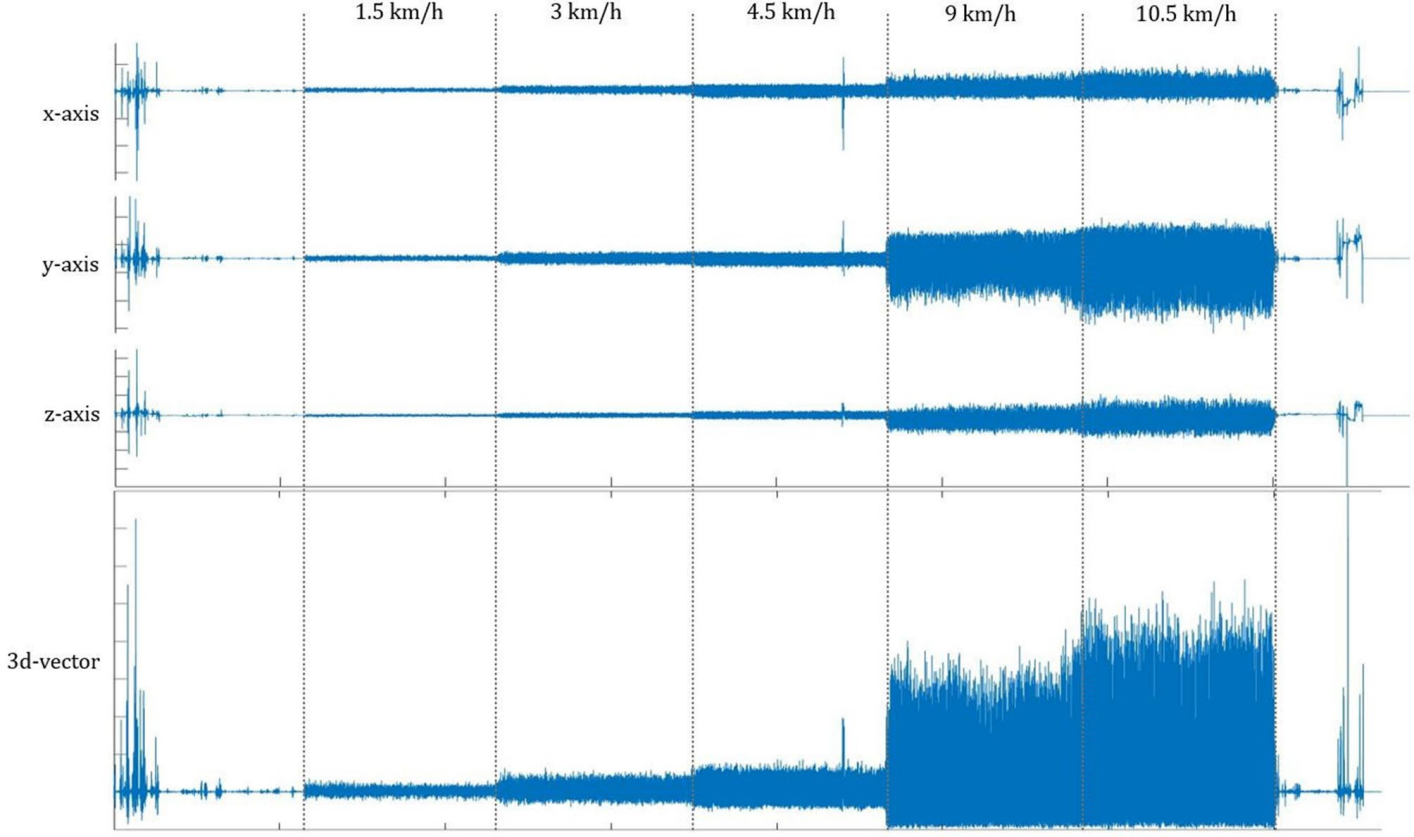
Example of Sartorio Xelometer data. Raw data recording of one subject with different speeds marked with dashed lines. x, y and z-axis recordings separately and a 3d-vector composed of the triaxial data. Further calculations are conducted using the 3d-vector.

Subjects wore three different accelerometers on their body during the exercise. ActiGraph GT3X+ and Sartorio Xelometer were worn on elastic belt on the hip on the right side of the body. Sartorio Xelometer (Supplementary Figure 4) is a novel tri-axial accelerometer with a raw acceleration data output (g) with a ± 8 g range, 0.0156 g resolution, 100 Hz sampling rate and a battery life of 21 consecutive days of measurement. No data processing takes place within the device and all data processing is done in MATLAB R2019a software. ActivPAL was worn on the left thigh. Manufacturer’s software was used to set up the device and determine step counts and EE estimates. For the EE estimation, activPAL uses the following equation: MET × h^−1^ = (1.4 × d) + (4–14) × (c/120) × d, where c = cadence (steps per minute) and d = activity duration (in hours)^34^. ActiGraph GT3X+ data was extracted with ActiLife v6.13.4 and step counts were determined using 1 s epochs and 100 Hz sampling rate to accurately define the counts for different speeds. For energy expenditure (METs), Freedson Adult (1998) cut points were used within the software (equation: MET Rate = 1.439008 + (0.000795 × CPM) where CPM = Counts per Minute)^35^. For activPAL, PALconnect v8.10.8.76 was used to set up the devices and extract the data and PALanalysis v8.11.2.54 to analyze the step counts and EE estimates (METhrs). MET-hours from activPAL were transformed into METs for comparisons. Only total EE for the recordings are available from ActiGraph’s ActiLife and activPAL’s s software, thus preventing comparison on 4-min interval level with the IC. Sartorio Xelometer data was extracted using Sartorio v18 software and detection algorithms provided by the manufacturer were run on MATLAB R2019a for step counts, step intensities and EE estimates (MET). The step detection algorithm creates a 3d-vector based on the recordings from the three axes (Fig. 3). When 3d-vector signal exceeds the certain threshold, this exceeding area is analyzed based on the peak amplitude, rising and declining time. The presented data have not been used in the development in the Sartorio step detection algorithm 1 or EE estimation algorithm or the software. The EE estimation algorithm for the Xelometer is based on the signal vector and results of Vähä-Ypyä et al.^36^, following the equation: VO_2_ = 7.920–0.0331*MAD (mg). MAD (mean amplitude deviation) was calculated with the following equation: $MAD = \frac{1}{N}\sum\nolimits_{i = j}^{j + N - 1} {\left| {r_{i} - R_{ave} } \right|,}$ where N is the number of samples in the epoch, j is the start of the epoch and R_ave_ is the mean resultant value. The conversion of VO_2_ from the Sartorio EE estimation equation to MET was done using the standard conversion factor (1 MET = 3.5 ml*kg^−1^*min^−1^). Data from the optimization cohort 1 were used to optimize the Sartorio Xelometer step detection algorithm. The step detection program was developed using a machine learning algorithm. The algorithm applied supervised learning and used the optimization cohort 1 data as the training data. The data included exact step counts obtained by the video analysis with four different walking/running speeds and a recorded 3d acceleration signal. These speeds were 1.5, 3, 9 and 10.5 km/h, respectively. The algorithm was developed using MathWorks Matlab R2019a. The machine learning algorithm applied following parameters related to the norm of the 3d acceleration signal: (I.) a threshold value for the 3d acceleration (the acceleration value needs be higher than the threshold to be accepted as the starting point of an acceleration peak to be analyzed). (II.) The maximum value of the 3d acceleration peak. (III.) The slope of the 3d acceleration peak. (IV.) The area of the 3d acceleration peak. (V.) The time difference between consecutive 3d acceleration peaks. The algorithm tested different threshold values for the given parameters. If the accelerations were acting in the predetermined ways (for example, an acceleration value should be higher than the corresponding threshold, the time difference shorter than the corresponding threshold etc.) related to the tested parameters they were accepted as step counts and the relative error comparing to the results obtained by the video analysis were calculated. Those parameter values that yielded the lowest relative errors were chosen to the final step count program (Supplementary Table 4).

Subjects in the validation cohort 2 (n = 19) completed a similar set of measurements with following exceptions. RMR and energy consumption were not measured since the optimization of the algorithm does not affect the EE estimation. The brisk walking speed of 6 km/h was added into the protocol.

MAPE percentages were calculated in all speeds between the actual (video) and accelerometer-estimated step counts with the following equation: $M\% = \left( {\frac{1}{n}\sum\nolimits_{t = 1}^{n} {\left| {\frac{{A_{t} - F_{t} }}{{A_{t} }}} \right|} } \right) \times 100.$

MAPE percentages of over 5 were considered as relevant disagreement^15,37^. For EE estimates only total EE for 20 min was used in the assessment. Data from the accelerometers and IC were transformed into METs for comparisons. To observe the similarity between video and accelerometer step counts, paired samples *t* test, linear regression and intraclass correlation were calculated and Bland–Altman plots drawn for step counts in different speeds. All statistical analysis was done, and figures generated using IBM SPSS Statistics version 26. P-values less than 0.05 were considered statistically significant. ICC over 0.90 was considered excellent, 0.75–0.90 good, 0.75–0.60 moderate and less than 0.60 as low. Results in the Tables 4 and 5 are represented as mean ± standard deviation.

## Conclusions

The Xelometer is a valid device for assessing step counts at different gait speeds. Its accuracy is comparable to widely used activPAL and different than ActiGraph GT3X+. The EE estimates of all three devices were inaccurate when compared with indirect calorimetry.

## Supplementary Information

Supplementary Information.
