# Supplementary material for: Step detection and energy expenditure at different speeds by three accelerometers in a controlled environment
Source: Sci Rep. 2021 Oct 8;11:20005. doi: 10.1038/s41598-021-97299-z (PMC8501125; doi:10.1038/s41598-021-97299-z)
Supplement: Supplementary file 1 — Supplementary Information. [file 41598_2021_97299_MOESM1_ESM.docx]

**Step detection and energy expenditure at different speeds by three accelerometers in a controlled environment**

Ville Stenbäck ^1,2^, Juhani Leppäluoto ^1^, Nelli Leskelä^1^, Linda Viitala^1^, Erkki Vihriälä^3^, Dominique Gagnon^1,4^, Mikko Tulppo^1^ and Karl-Heinz Herzig ^1,5^

Supplementary Table 1. Means ± standard deviations and 95% limits of agreement for the Bland-Altman plots in each speed and device in the optimization cohort 1.

|  |  |  | **95% limits of agreement** | |
| --- | --- | --- | --- | --- |
| **Sartorio** | **Speed (km/h)** | **Mean ± std.dev.** | **Upper (+1.96 SD)** | **Lower (-1.96 SD)** |
|  | **1.5** | -1.046 ± 0.387 | -0.284 | -1.801 |
|  | **3** | -1.260 ± 0.558 | -0.166 | -2.354 |
|  | **4.5** | -2.012 ± 1.271 | 0.478 | -4.503 |
|  | **9** | -1.355 ± 1.407 | 1.402 | -4.113 |
|  | **10.5** | -1.337 ± 0.666 | -0.030 | -2.644 |
|  | **total** | -1.334 ± 0.980 | 0.587 | -3.256 |

Supplementary table 2. Means ± standard deviations and 95% limits of agreement for the Bland-Altman plots in each speed for the Sartorio Xelometer EE estimation.

Supplementary Table 3. Means ± standard deviations and 95% limits of agreement for the Bland-Altman plots in each speed and device in the validation cohort 2.

Supplementary Table 4. The parameters for the Sartorio Xelometer step detection program on the low acceleration area. The program analyzes the length of the norm of the 3d acceleration signal.

| **Parameter** | **Value** | **Unit** |
| --- | --- | --- |
| 1. Threshold | 9.957 | m/s^2^ |
| 2. The maximum value of the 3d acceleration peak | 10.644 | m/s^2^ |
| 3. Slope | 44.145 | m/s^3^ |
| 4. The area of the acceleration peak | 0.7848 | m/s |
| 5. The time difference between consecutive acceleration peaks | 0.510 | s |


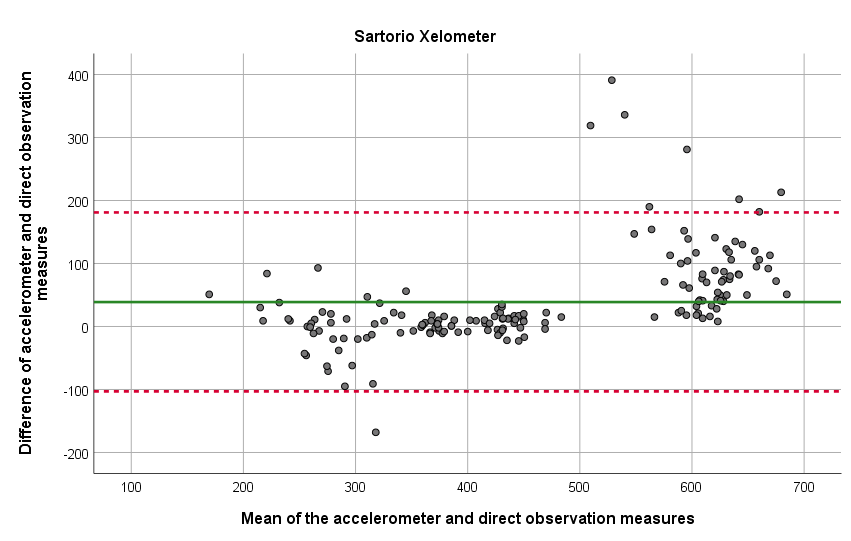


Supplementary Figure 1. Bland-Altman plot of steps detected with Sartorio Xelometer compared to direct observation in the optimization cohort 1. Differences and means were calculated for all speeds (4-minute intervals) separately and plotted on the same graph. Solid lines: means of difference. Dashed lines; 1.96 SD of difference.


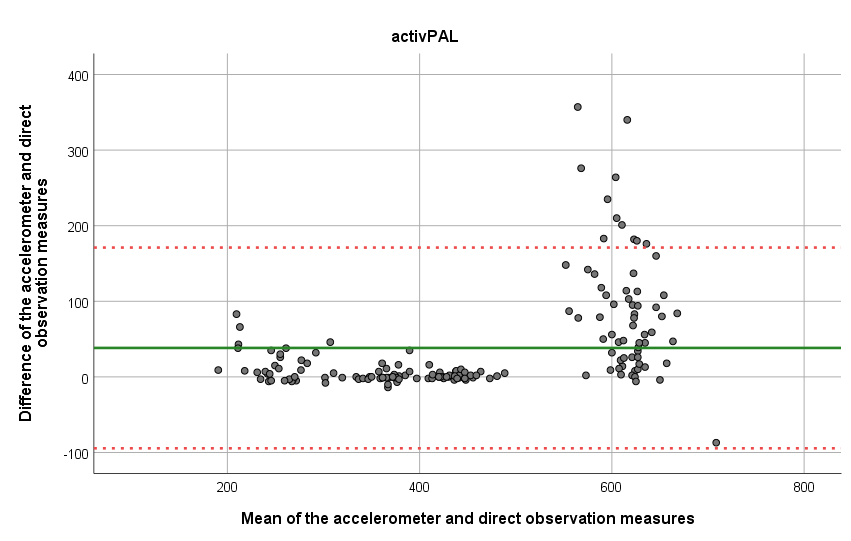


Supplementary Figure 2. Bland-Altman plot of steps detected with activPAL compared to direct observation in the optimization cohort 1. Differences and means were calculated for all speeds (4- minute intervals) separately and plotted on the same graph. Solid lines: means of difference. Dashed lines; 1.96 SD of difference.


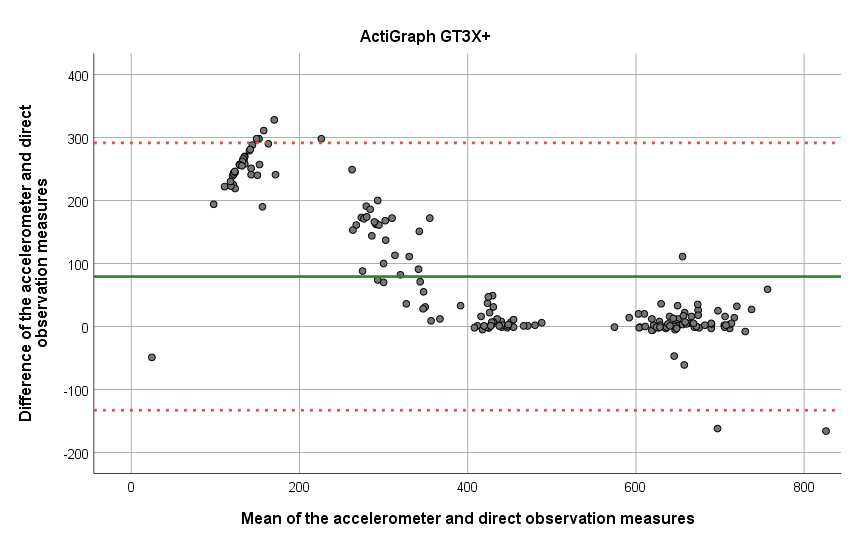


Supplementary Figure 3. Bland-Altman plot of steps detected with ActiGraph GT3X+ compared to direct observation in the optimization cohort 1. Differences and means were calculated for all speeds (4-minute intervals) separately and plotted on the same graph. Solid lines: means of difference. Dashed lines; 1.96 SD of difference.


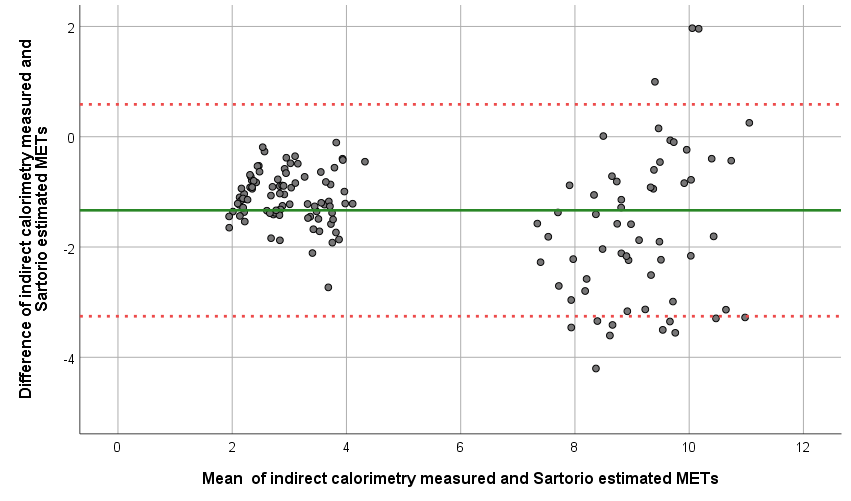


Supplementary Figure 4. Bland-Altman plot of MET estimated with Sartorio Xelometer and indirect calorimetry measured MET in the optimization cohort 1. Differences and means were calculated for all speeds (4-minute intervals) separately and plotted on the same graph. Solid lines: means of difference. Dashed lines; 1.96 SD of difference.


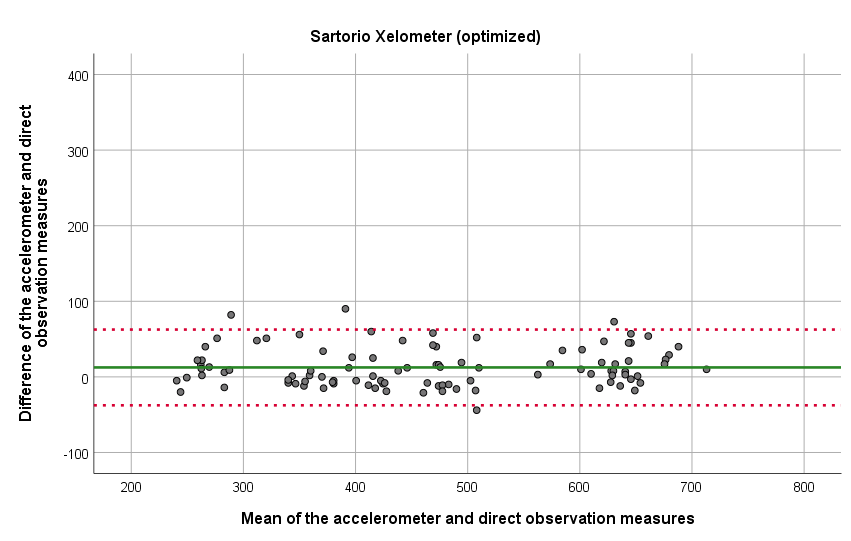


Supplementary Figure 5. Bland-Altman plot of steps detected with Sartorio Xelometer compared to direct observation in the validation cohort 2. Differences and means were calculated for all speeds (4-minute intervals) separately and plotted on the same graph. Solid lines: means of difference. Dashed lines; 1.96 SD of difference.


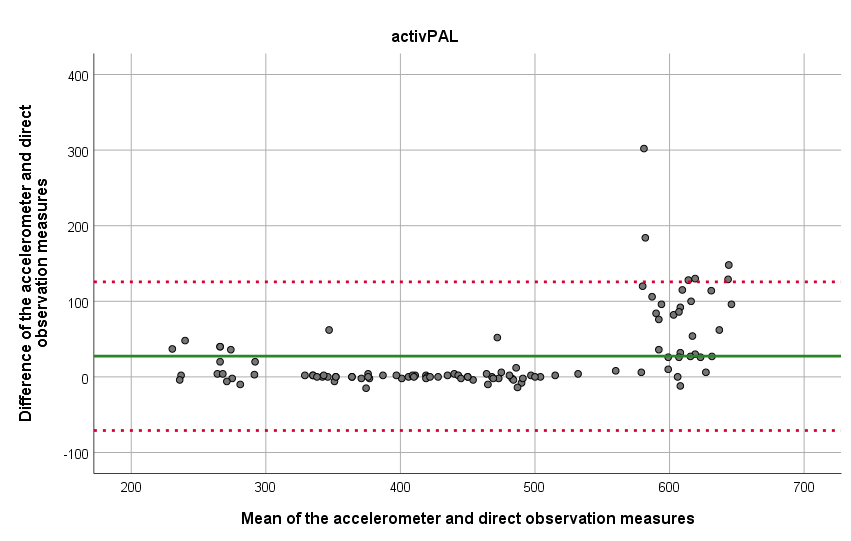


Supplementary Figure 6. Bland-Altman plot of steps detected with activPAL compared to direct observation in the validation cohort 2. Differences and means were calculated for all speeds (4-minute intervals) separately and plotted on the same graph. Solid lines: means of difference. Dashed lines; 1.96 SD of difference.


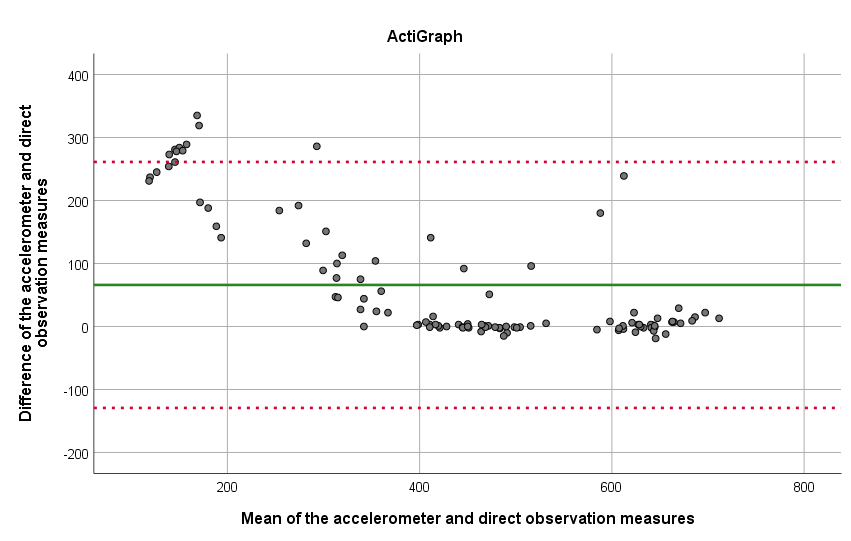


Supplementary Figure 7. Bland-Altman plot of steps detected with ActiGraph GT3X+ compared to direct observation in the validation cohort 2. Differences and means were calculated for all speeds (4-minute intervals) separately and plotted on the same graph. Solid lines: means of difference. Dashed lines; 1.96 SD of difference.


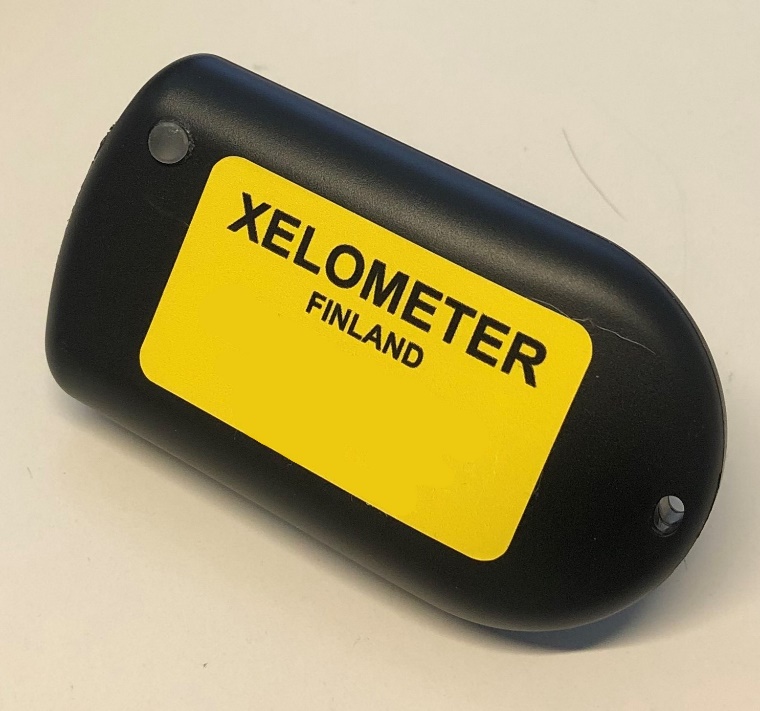


Supplementary Figure 8. The of view of Sartorio Xelometer. Dimensions are 83mm x 48mm x 15mm.
